# Comprehensive Comparison of Baculoviral and Plasmid Gene Delivery in Mammalian Cells

**DOI:** 10.3390/v16030426

**Published:** 2024-03-10

**Authors:** Maria Toth, Manuel Reithofer, Gregory Dutra, Patricia Pereira Aguilar, Astrid Dürauer, Reingard Grabherr

**Affiliations:** 1 Department of Biotechnology, University of Natural Resources and Life Sciences Vienna, 1190 Vienna, AT, Austria; 2 Austrian Centre of Industrial Biotechnology (ACIB), 1190 Vienna, AT, Austria

**Keywords:** BacMam, baculovirus, transfection, transduction, protein production, expression enhancement, stable cell lines

## Abstract

(1) Recombinant protein production in mammalian cells is either based on transient transfection processes, often inefficient and underlying high batch-to-batch variability, or on laborious generation of stable cell lines. Alternatively, BacMam, a transduction process using the baculovirus, can be employed. (2) Six transfecting agents were compared to baculovirus transduction in terms of transient and stable protein expression characteristics of the model protein ACE2-eGFP using HEK293-6E, CHO-K1, and Vero cell lines. Furthermore, process optimization such as expression enhancement using sodium butyrate and TSA or baculovirus purification was assessed. (3) Baculovirus transduction efficiency was superior to all transfection agents for all cell lines. Transduced protein expression was moderate, but an 18-fold expression increase was achieved using the enhancer sodium butyrate. Ultracentrifugation of baculovirus from a 3.5 L bioreactor significantly improved the transduction efficiency and protein expression. Stable cell lines were obtained with each baculovirus transduction, yet stable cell line generation after transfection was highly unreliable. (4) This study demonstrated the superiority of the BacMam platform to standard transfections. The baculovirus efficiently transduced an array of cell lines both transiently and stably and achieved the highest efficiency for all tested cell lines. The feasibility of the scale-up of baculovirus production was demonstrated and the possibility of baculovirus purification was successfully explored.

## 1. Introduction

The production of recombinant proteins is the greatest economic factor in the biotechnological industry. The efficient production of biopharmaceuticals in mammalian cells often relies on the use of stable cell lines. Their generation, however, requires time-consuming and cumbersome establishment steps, resulting in higher risks and costs. Transfection with plasmids, with the integration of a target expression cassette, and subsequent clone selection are the standard methods for the production of complex, soluble proteins like antibodies. For the expression of even more complex multisubunit structures like virus-like particles (VLP) for vaccines or adeno-associated virus (AAV) for gene therapy, the classical concept of transient plasmid transfection is adopted. These processes are sometimes very inefficient and often depend on a production cell line [1,2,3]. Moreover, the production of AAVs requires a sophisticated combination of different plasmids at varying ratios to achieve the highest possible yield. Regardless of the degree of standardization, these approaches are prone to variations and a stable reproducible process is still missing.

In recent years, an alternative to conventional procedures has gained and regained popularity. The insect-specific baculovirus is not only an attractive tool for protein expression in insect cells but also for gene delivery in various mammalian cell lines. The baculovirus was implemented for expressing bionanoparticles [4,5] and vaccines, either in the form of VLPs and recombinant subunits or as gene therapy vectors such as AAVs [6,7,8,9,10]. The natural hosts of the baculovirus are insects, more specifically the phylum of Arthropods. It contains a circular, double-stranded DNA genome with a size of up to 180 kbp [11]. Despite the fact that baculoviruses are able to enter mammalian cells and transfer genetic material, they are not capable of replicating in these hosts and consequently do not require high biosafety level laboratories [12,13]. Therefore, sophisticated approaches utilizing the virus as a vehicle for vector DNA transduction were developed, and the BacMam platform was established in the early 2000s. Various studies showed that when the gene of interest is controlled by a mammalian promoter, substantial target protein expression can be achieved. Furthermore, it could be shown that a high variety of mammalian cell lines could be targeted, but with different efficiencies and protein yields [14,15,16]. The most important feature of the BacMam system is its capacity to harbor large DNA fragments within the viral genome facilitating the expression of entire protein complexes. The pioneering work in this field was published by the group of Imre Berger in the early 2000s. They described the expression of protein complexes using just one baculovirus construct, which was implemented by the Multibac system [17,18,19]. This method was based on Cre/loxP recombination for the efficient cloning of large DNA constructs. Several other commercially available approaches, such as the Tn7L/Tn7R system for efficient transposition of the plasmid DNA into the baculovirus genome were developed around that time; for a review, see [20,21].

Another feature, similar to plasmid-based expression in mammalian cells is the possibility to enhance expression levels with the addition of histone deacetylase inhibitors [22]. Foreign transfected or transduced DNA is silenced by modification via host histone deacetylases as a self-defense mechanism in mammalian cells [23,24,25]. Previous reports already demonstrated the effect of trichostatin A (TSA) or sodium butyrate, which are potent histone deacetylase inhibitors, on different cell types. The addition of these compounds leads to increased expression levels after transfection and even more prominent expression upregulation after baculoviral transduction [26,27]. The increased popularity combined with the cost-effectiveness, robust transduction processes, and straightforward scalability has led to the implementation of the BacMam platform in large-scale production processes for recombinant proteins [28,29]. However, in some cases, the generation of stable cell lines is often the preferred method of choice. Additionally, for membrane protein interaction studies, a stable cell line with robust surface expression is an indispensable prerequisite. Albeit the ability of the BacMam system to transduce a variety of different mammalian cell lines, it is commonly believed that it is not suitable for stable cell line generation [21]. Yet, an early report described the capability of baculoviruses to be a powerful tool for the establishment of stable cell lines. The group around Thomas A. Kost demonstrated the potential of genome integration with the BacMam system in the late 90s with an adherent CHO cell line [27].

In this work, we intended to compare baculovirus transduction with conventional, cell-permeating transfection agents in terms of efficiency and productivity on a transient basis as well as the feasibility of random genome integration to generate stable cell lines. Furthermore, we included a large-scale baculovirus preparation in a bioreactor to underline the potential for upscaling and industrial recombinant protein production.

## 2. Materials and Methods

### 2.1. Cell Culture

Adherent CHO-K1 and Vero CCL-81 cells were grown in DMEM:F12 (1:1) (Gibco) supplemented with 2 mM L-Glutamine (Gibco) and 5% FBS (Gibco) at 37 °C and 8% CO_2_. Suspension HEK293-6E were grown in FreeStyle^TM^ F17 (Gibco) supplemented with 4 mM L-Glutamine (Gibco) and 0.1% Pluronic F-68 (Gibco) at 37 °C, 8% CO_2_, and 120 rpm.

### 2.2. Baculovirus Production

ACE2-GFP was amplified by PCR with the primers 23.29 and 23.30 from pcDNA3.1-ACE2-GFP. pcDNA3.1-ACE2-GFP was a gift from Utpal Pajvani (Addgene plasmid #154962; http://n2t.net/addgene:154962 accessed on 7 March 2024; RID: Addgene_154962) [30]. The backbone pACEBac-1 (Geneva Biotech, Pregny-Chambésy, Switzerland) was amplified with 23.31 and 23.32 (Table 1) and the two fragments were combined using the golden gate cloning method with the enzyme PaqCI. Subsequently, the plasmid underwent a transformation into EMBacY-competent cells (Geneva Biotech). The resulting bacmid was then introduced into Sf9 cells using the FuGENE^®^ HD transfection reagent (Promega, Madison, WI, USA), in accordance with the guidelines supplied by the manufacturer. Following two passages in shaking flasks, a working stock was produced and assessed for concentration using TCID50. The cultures were performed for 72 h or until the viability dropped to 70%.

A large batch of baculovirus was produced in a Bioflo reactor and subsequently purified using ultracentrifugation (Dutra et al., under review, [31]). Briefly, a 3.5 L final volume was produced in a 5 L BioFlo 320 stirred tank bioreactor system (Eppendorf). Cells were grown to 3 × 10^6^ cells/mL in 2 L and, prior to infection with an MOI of 0.01, the suspension was diluted with fresh medium to a density of 2 × 10^6^ cells/mL. The infection lasted for 96, and thereafter the supernatant was harvested and filtered through a 0.8 µm filter (Sartorius).

### 2.3. Ultracentrifugation of Baculorivus

Density gradient ultracentrifugation was performed using a PKII continuous flow ultracentrifuge (Alfa Wassermann, MX IJsselstein, The Netherlands) according to [31]. The separation of the particles was achieved by using an 800 mL core filed with a sucrose gradient, composed of 200 mL of each gradient solution (50% and 45% sucrose, % by weight) and 400 mL of buffer (50 mM HEPES, 150 mM NaCl, pH 7.2). A linear sucrose gradient was formed and reoriented vertically during rotor acceleration. After the centrifuge reached the target speed of 35,000 rpm, 950 mL of clarified supernatant was pumped into the centrifuge at a 1 L/h flow rate. A banding time of 45 min was used. After the banding time, the centrifuge was decelerated and stopped, allowing the gradient to reorient horizontally. After that, the gradient was collected from the ultracentrifuge in 30 mL fractions.

### 2.4. Transfection

For transfection experiments, the plasmid pcDNA3.1-ACE2-GFP encoded a fusion protein of the membrane receptor ACE2 with eGFP under the CMV promoter and enhancer. Transient transfections were performed in 24-well plates and expression was allowed for 48 h. Adherent cells were seeded the day before transfection with 50,000 cells per well, whereas suspension HEK293-6E was seeded with 100,000 cells per well directly. Transfection was performed with polyethylenimine 25 kDa (PEI) (Polysciences), Lipofectamine 3000 (Thermo Fisher Scientific), FuGENE^®^ HD (Promega), Attractene (Qiagen), and PolyFect (Qiagen) according to the manufacturer’s instructions. Opti-MEM (Gibco) was used if required. Calcium phosphate transfections were conducted with 2.5 M calcium chloride (Sigma-Aldrich, St. Louis, MO, USA) and 2X HEPES buffered saline (HBS) (0.28 M NaCl, 0.05 M HEPES (Sigma-Aldrich), 1.5 mM Na_2_HPO_4_, pH 7.05). Therefore, 0.5 µg DNA was added to 20 µL RO-H_2_O and 3 µL of 2.5 M calcium chloride. A total of 24 µL of mM HBS was added dropwise during vortexing of the mixture.

Transfections for stable cell line generation were performed in 6-well plates with 500.000 cells per well for adherent cells (MOI 100) and in 24-well plates for suspension HEK293-6E cells (MOI 10) with 100.000 cells per well. After 48 h, the cells were split 1:2, and fresh medium supplemented with 500 µg/mL G418 (Gibco) was added for selection. Three weeks after transfection, the cells were analyzed. 

### 2.5. Transduction

Transduction experiments were performed in 24-well plates. Baculovirus preparations in insect medium were added to the cultures at indicated MOIs. After 16 h of incubation, a full medium change was performed and expression was allowed for 48 h starting at initial transduction. After 48 h, the cells were split 1:2, and fresh medium supplemented with 500 µg/mL G418 (Gibco) was added for selection. Two weeks after transduction, the cells underwent a cell-sorting step. 

### 2.6. Viability and Fluorescence Measurements

Adherent and suspension cells were harvested by collecting the medium, followed by incubation of the cells with 100 µL trypsin at 37 °C and inactivation of trypsin with 400 µL DMEM:F12 (1:1) (Gibco, Waltham, MA, USA) supplemented with 2 mM L-Glutamine (Gibco) and 5% FBS (Gibco). The cell suspension was added to the previously collected medium. Viability was measured with ViCELL XR Cell Analyzer (Beckman Coulter, Brea, CA, USA). Samples for flow cytometry were centrifuged at 400× *g* for 5 min. The supernatant was removed and the pellet was washed with 1 mL of PBS, followed by a second centrifugation step at 400× *g* for 5 min. The supernatant was again discarded and the cell pellet was resuspended in 200 µL PBS (Corning, Corning, NY, USA). Flow cytometric analysis was performed using CytoFLEX S (Beckman Coulter), and the data were evaluated using Kaluza 2.1 software (Beckman Coulter). gMFI was calculated as the Nth root of the product of all intensities obtained by every single cell.

The evaluation of stable transfection of adherent cells was performed by Leica DM IL LED microscope. Therefore, cells were fixated with 4% paraformaldehyde (VWR) and subsequently washed with PBS three times. Each well was assessed for outgrown fluorescent colonies. A colony of more than 15 fluorescent cells was counted as a successful stable transfection.

### 2.7. Cell Sorting

**Figure A1:**
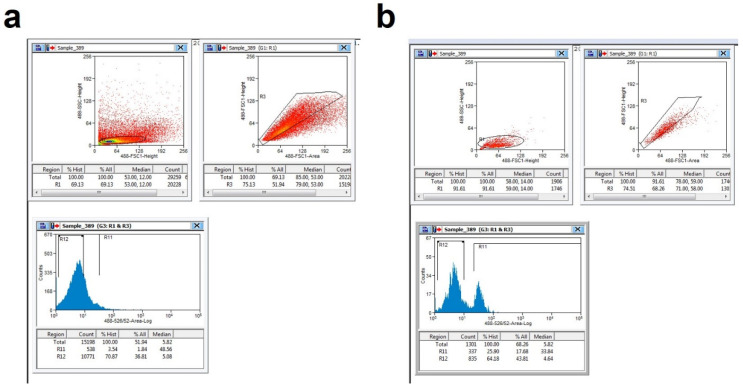
Stringent sorting strategy of HEK (**a**) and CHO (**b**) cells after 2 weeks of selection.

**Figure A2:**
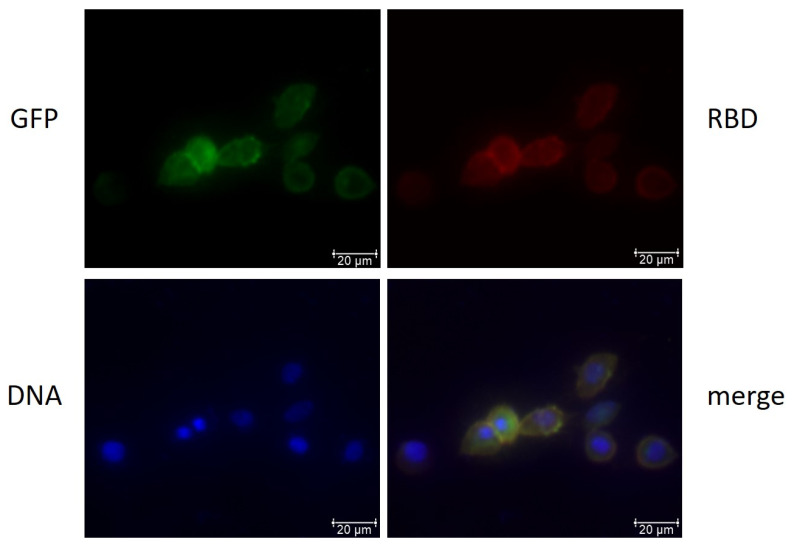
Stringent immunofluorescent staining of transduced CHO-K1 cells. Cells were transduced with baculovirus and expression was allowed for 48 h. Thereafter, cells were fixated with 4% PFA, and ACE2 was detected with His-tagged RBD (Klausberger et al. 2021 and 2022). The Histag was detected with mouse Anti Histidine Tag antibody (BioRad). As a secondary antibody to detect the mouse Anti Histidine Tag antibody, a goat anti-mouse* Phycoerythrin antibody (Sigma-Aldrich) was used.

After two weeks of selection with 500 µg/mL G418 (Gibco), the cells were harvested, washed, and resuspended in cold PBS. Thereafter, GFP-positive cells were sorted with a BC MoFlo Astrios EQ (Beckmann Coulter) into a 24-well plate with 50.000 cells per well in 500 µL (Figure A1) and tested for ACE2 expression (Figure A2). 

### 2.8. Data Analysis

Statistical tests were performed with GraphPad Prism 10 software using one-way ANOVA with Tukey’s or Dunnett’s post-test or Student’s *t*-test. The significance level was set as *p* ≤ 0.05.

## 3. Results

### 3.1. Transient Transfection and Transduction

**Figure 1:**
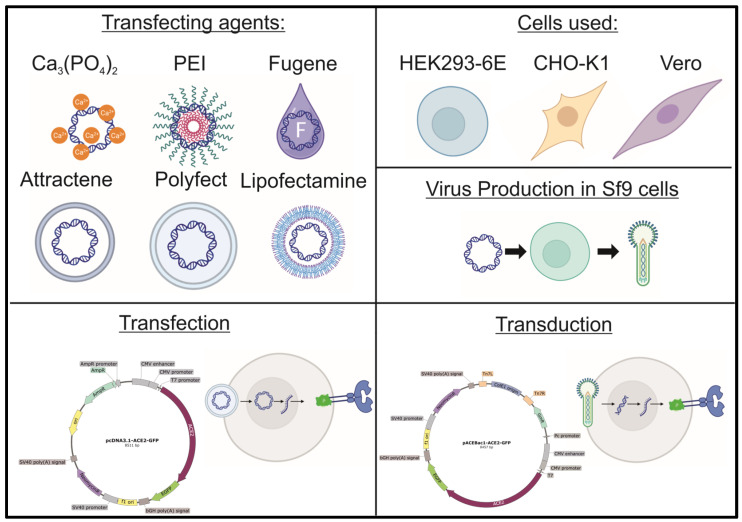
Workflow of the comparison of six transfecting agents to baculovirus transduction in three different cell lines.

In this study, six conventional transfection methods were compared to baculovirus transduction in terms of transfection efficiency, protein expression levels, and the efficacy of generating stable cell lines (Figure 1). Our reporter gene encoded a fusion protein consisting of the human ACE2 receptor, representing a complex, glycosylated protein and the enhanced green fluorescent protein (ACE2-eGFP).

#### 3.1.1. MOI Titration

**Figure 2:**
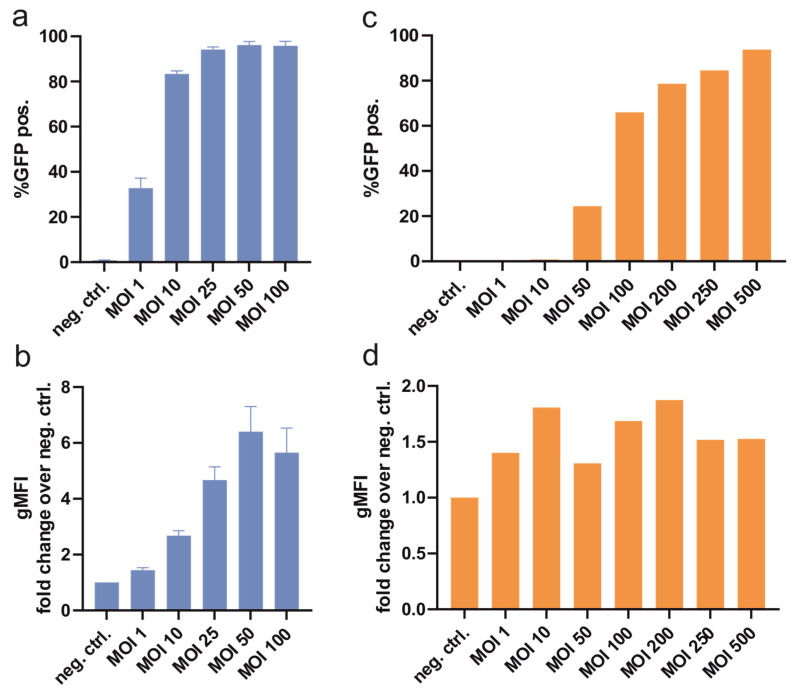
Determination of optimal transduction settings by baculovirus multiplicity of infection (MOI) titration. (**a**) Transduction efficiency expressed as a percentage of fluorescent HEK293-6E cells and (**b**) protein expression level measured as cellular fluorescence. Each bar represents four to five independent experiments. (**c**) Transduction efficiency of CHO-K1 cells and (**d**) cellular fluorescence resulting from a single experiment.

As a pre-experiment, optimal transduction settings were evaluated by determining the optimal multiplicity of infection (MOI) of baculoviruses with the suspension cell line HEK293-6E and the adherent cell line CHO-K1. With increasing MOI, higher transduction efficiencies were observed, as shown in Figure 2a,b. At 96%, the highest efficiency was achieved in HEK293-6E with MOI 50. Increasing expression levels were visible for HEK293-6E up to an MOI of 50. For this cell line, MOI 10, 25, and 50 were chosen for further experiments.

With the CHO-K1 cell line, higher MOIs were necessary to achieve acceptable transduction levels (Figure 2c), yet increased protein expression per cell was not observed with higher MOIs (Figure 2d). For CHO-K1, the following experiments were conducted with MOI 50, 100, and 250. 

#### 3.1.2. Recombinant Protein Expression after Transduction and Transfection

**Figure 3:**
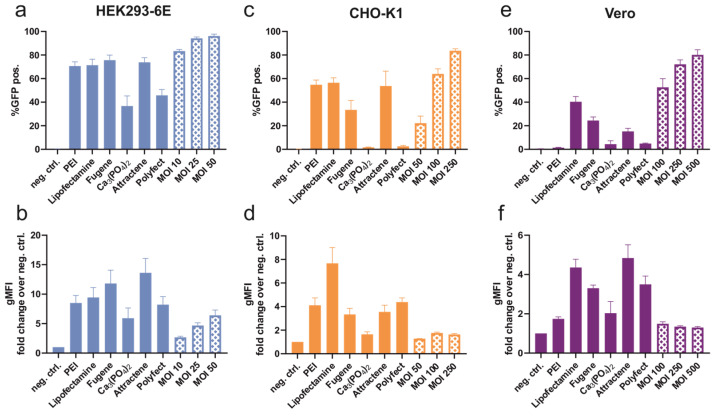
Comparison of GFP expression between transfecting agents PEI, Lipofectamine, Fugene, calcium phosphate, Attractene, and Polyfect and baculovirus transduction at different MOIs with the three cell lines HEK293-6E (**a**,**b**), CHO-K1 (**c**,**d**), and Vero (**e**,**f**). (**a**,**c**,**e**) Transfection efficiency is assessed as the number of cells that exhibit fluorescence (%GFP pos.) and (**b**,**d**,**f**) GFP expression is measured as the geometric mean of fluorescence intensity (gMFI), calculated as a fold change in fluorescence over the negative control. Each bar represents five to six independent experiments and was evaluated using one-way ANOVA with Tukey’s post-test.

HEK293-6E turned out to be the most susceptible cell line to both transfection and transduction (Figure 3a). Over 70% of cells expressed ACE2-eGFP after transfection with PEI, Lipofectamine, Fugene, and Attractene. Of note, 96% of the baculovirus-treated cells (with MOI 50) expressed the recombinant protein, demonstrating a significant increase in efficiency compared to all transfecting agents. Transduction with MOI 25 showed significantly superior efficiency than all transfecting agents except Fugene. Although transduction with MOI 10 showed better efficiency than transfection, no significant difference could be detected between the four best-performing transfecting agents. Within the different MOIs, no significant difference was detected. Figure 3b shows the mean fluorescence that was exhibited per cell. Transfecting agents resulted in higher expression levels compared to baculovirus transduction. The lowest expression was achieved using calcium phosphate, followed by Polyfect, PEI, Lipofectamine, and Fugene, and the highest expression was detected using Attractene. Over a 13-fold increase in fluorescence was achieved with Attractene. Baculovirus transduction resulted in a lower protein expression compared to transfection, yet an increment in protein expression was observed with increasing MOI. Functionality of the ACE2 receptor, expressed by transduced cells, was confirmed using immunofluorescence (Figure A2). 

Adherent CHO-K1 (Figure 3c) exhibited over 50% fluorescent cells after transfection with PEI, Lipofectamine, and Attractene. In total, 84% of the cells were transduced using an MOI of 250 of baculovirus, which was significantly more efficient compared to all other transfecting agents except for Lipofectamine. GFP expression levels per cell depicted in Figure 3d showed the highest expression after Lipofectamine transfection with an over 7-fold increase in fluorescence intensity. Medium expression levels were accomplished by using PEI, Fugene, Attractene, and Polyfect. Baculovirus transduction resulted in lower increases in fluorescence compared to the other methods tested. 

Adherent Vero cells were generally less prone to transfection compared to the other cell lines (Figure 3e). The most effective transfecting agent for this cell line, Lipofectamine, reached 40% of the cells followed by Fugene with around 25% and Attractene with 15%. Transduction with baculovirus resulted in the highest efficiency with 80% of the cells exhibiting an expression of GFP. Significantly higher efficiencies were seen for transduction with MOI 250 and 500, whereby no significant differences were observed between these two MOIs. The highest protein production per cell was achieved with Attractene followed by Lipofectamine, Polyfect, and Fugene (Figure 3f). Baculovirus transduction again resulted in a lower increase in fluorescence compared to the other methods. 

#### 3.1.3. Cell Viability after Transduction and Transfection

**Figure 4:**
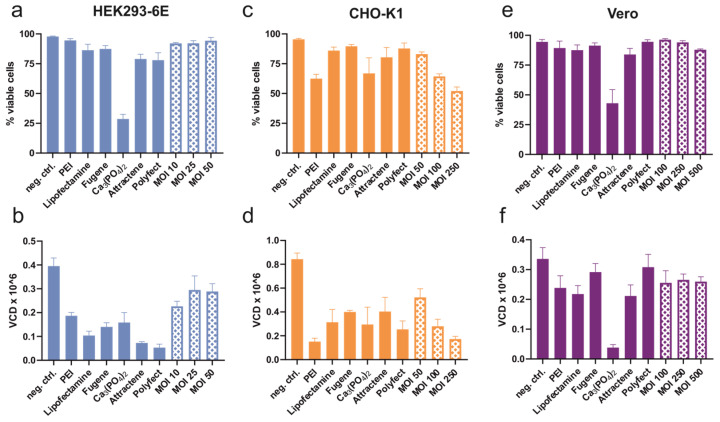
Comparison of viability data between the indicated transfecting agents and baculovirus transduction at different MOIs with the three cell lines HEK293-6E (**a**,**b**), CHO-K1 (**c**,**d**), and Vero (**e**,**f**). (**a**,**c**,**e**) Viability expressed as a percentage of viable cells and (**b**,**d**,**f**) viable cell density (VCD) measured with the ViCell XR Cell Analyzer. Each bar represents three to five independent experiments and was evaluated using one-way ANOVA with Tukey’s post-test.

Maintenance of high viability is crucial during recombinant protein expression processes as well as for stable cell line generation. Figure 4a depicts the viability of HEK293-6E. After transfection with PEI, Lipofectamine and Fugene the viability was above 85%, followed by Attractene and Polyfect exhibiting viabilities of above 75%. Calcium phosphate resulted in a decrease in viability to 29%. The baculovirus transductions displayed the highest viability of about 92%. The viable cell density (VCD) (Figure 4b) was reduced for all transfecting agents by more than 50%, resulting in VCDs of less than 0.2. Baculovirus transduction showed the highest viability and VCD, which was maintained above 0.2. A higher MOI did not result in a lower viability or VCD. Baculoviral transduction with MOI 25 or MOI 50 showed no significant decline in VCD compared to the negative control, yet a significant increase was observed compared to all transfecting agents. 

In CHO-K1, viability of less than 70% and reduced VCD was observed when using PEI, calcium phosphate, or baculovirus of MOI 100 or 250 (Figure 4c,d). Baculovirus at an MOI of 50 showed the highest VCD and was the only experimental setup for CHO-K1 where no significant differences from the negative control could be determined. Furthermore, the VCD was not reduced to less than 50% of the negative control.

Vero cells exhibited high viability for all methods tested, except for calcium phosphate (Figure 4e). Baculovirus transduction was the only method to result in an efficiency of above 50% (Figure 3e), yet viability was high. VCD was maintained above 50% of the negative control for all settings, except for calcium phosphate (Figure 4f). 

#### 3.1.4. Enhancement of Transduced Protein Expression

In our experiments, baculovirus gene delivery was more efficient compared to transfection with various agents, yet the expression per cell was lower. It was previously shown that chemicals such as sodium butyrate or trichostatin A (TSA) increase recombinant protein expression from viral DNA by inhibiting the deacetylation of histones [26,27]. The following experiments were conducted using the suspension HEK293-6E cell line, as it is not only the most susceptible cell line to baculovirus transduction, resulting in the highest transduction efficiency and protein expression while maintaining high cell viability, but is also used in many production processes. An MOI of 10 was chosen to demonstrate possible enhancing effects. 

**Figure 5:**
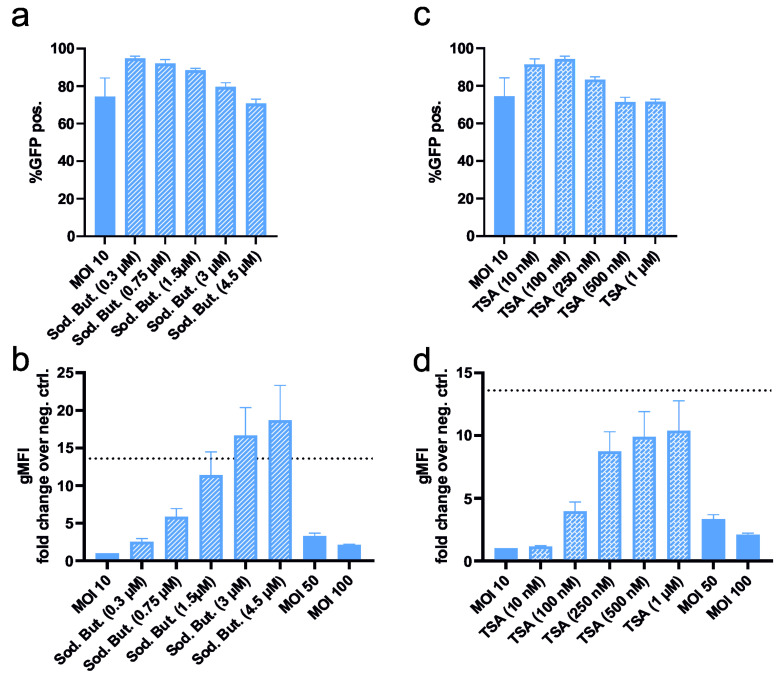
Baculovirus transduction of HEK293-6E at MOI 10 with an increasing concentration of sodium butyrate and trichostatin A (TSA). (**a**) The impact of increasing sodium butyrate concentrations on transduction efficiency and (**b**) GFP expression is assessed. (**c**) Transduction efficiency and GFP expression (**d**) with varying TSA concentrations are shown. GFP expression is measured as geometric mean fluorescent intensity (gMFI) in relation to the autofluorescence of the cell. Each bar represents three independent experiments and was evaluated using one-way ANOVA with Dunnett’s post-test. The dotted line in (**b**,**d**) represents expression levels achieved with Attractene transfection.

Figure 5a shows an increase in transduction efficiency when sodium butyrate was added. The number of GFP-positive cells was not affected to a high degree. However, as seen in Figure 5b, an increase in recombinant protein expression with increasing sodium butyrate concentrations could be achieved, resulting in an increment of over 18-fold between 4.5 and 0 µM sodium butyrate. Sodium butyrate concentrations of 1.5 to 4.5 µM demonstrated significant increases in fluorescence compared to MOI 10. Of note, the two highest sodium butyrate concentrations already exhibited a toxic effect on the cells. Apart from that, increasing the MOI of transduction did not result in a significant increase in fluorescence, and MOI 100 showed a reduction in gMFI compared to MOI 50. 

The addition of TSA also did not lead to an increase in transduction efficiency, but increasing TSA concentrations resulted in higher cellular fluorescence (Figure 5c,d). A total of 250 nM to 1 µM TSA resulted in significant increments in recombinant protein production compared to MOI 10. A TSA concentration of 1 µM led to a 10-fold higher GFP expression compared to no chemical added (MOI 10) but signs of toxicity were observed. 

#### 3.1.5. Transduction with Purified Baculovirus Produced on an Industrial Scale

**Figure 6:**
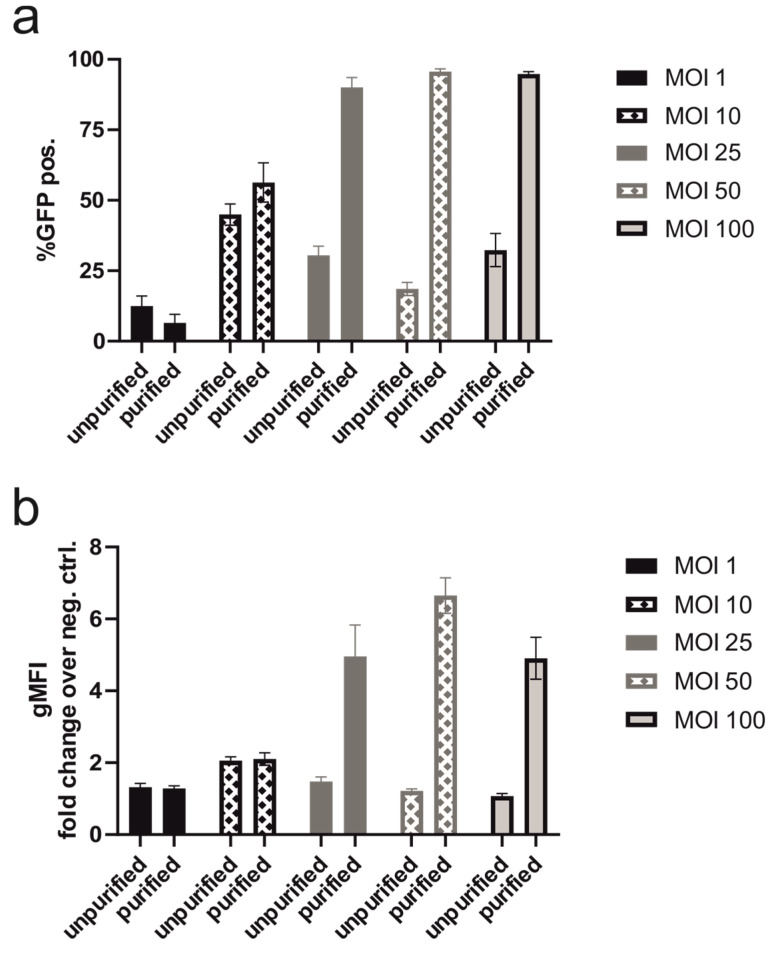
Comparison of transduction of HEK293-6E at varying MOIs with unpurified and purified baculovirus based on (**a**) transduction efficiency expressed as % GFP positive cells and (**b**) GFP expression assessed as geometric mean fluorescent intensity (gMFI) normalized to the autofluorescence of the cell. Each bar represents three independent experiments and was evaluated using Student’s *t*-test.

For the production of biopharmaceuticals via transient expression, upscaling is an important parameter. All experiments shown above were conducted with an unpurified baculovirus stock produced in small shake flasks. To test the scalability, the baculovirus production was performed in a bioreactor with 3.5 L of insect cell suspension. In the reactor, the cells were cultivated at higher densities compared to shaking flasks, which led to increased cell lysis during the whole process. The viability at the end was around 55% which was lower than in the shaking flasks, where the production was stopped at 70% viability. Our goal was to increase transduction efficiency by purifying the baculovirus. Ultracentrifugation removes toxic compounds from the insect cell culture supernatant and empty viral capsids. Figure 6 shows the comparison in transduction efficiencies between unpurified and purified viral preparation after bioreactor production at the indicated MOIs. Above MOI 10, transduction efficiency and GFP production were significantly increased with purified compared to unpurified baculovirus (Figure 6a), and no significant differences in transduction efficiency could be observed with increasing MOI of purified baculovirus (MOI 25 to 100). Figure 6b demonstrates significant increases in GFP expression by increasing MOI of purified baculovirus while the opposite effect was shown with unpurified baculovirus. 

### 3.2. Stable Transfection and Transduction

**Figure 7:**
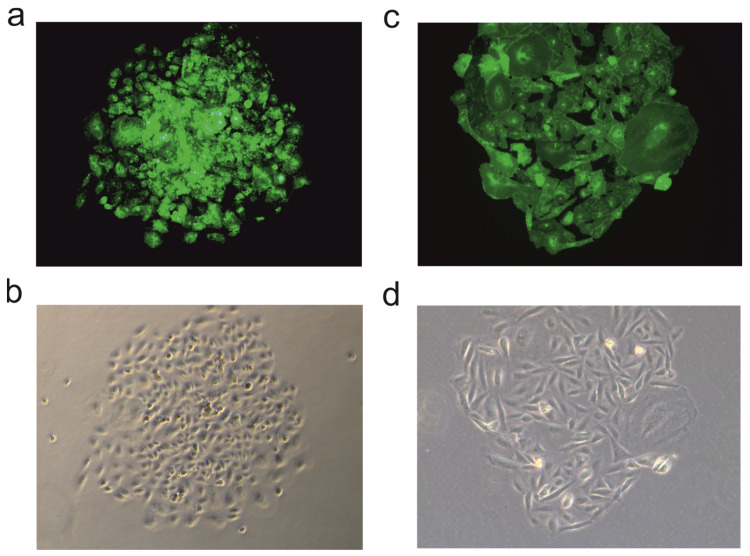
Depiction of a fluorescent colony that demonstrated stable cell line generation. The upper side shows fluorescence microscopy while the lower images depict the confocal microscopy image of the same colony. (**a**,**b**) Image of a stably transfected CHO-K1 colony using the transfecting agent Lipofectamine while (**c**,**d**) show a CHO-K1 colony transduced with Attractene.

We compared the feasibility of transfection agents and baculovirus transduction for random genome integration of a target gene. The successful generation of a stable cell line was defined as fluorescent cell colonies after 3 weeks of antibiotic selection pressure. Adherent cells were examined by fluorescence microscopy (Figure 7). Suspension cells, HEK293-6E, were measured by flow cytometry after 3 weeks of cultivation.

As shown in Table 2, in HEK293-6E only with Attractene transfection and baculovirus transduction, stable cell populations could be generated and cells exhibited growth during these 3 weeks. For CHO-K1, transfecting agents such as calcium phosphate and Polyfect, which showed low transfection efficiencies in transient expression experiments (Figure 3c), were not able to result in stable cell lines. On the other hand, PEI, Lipofectamine, and Attractene demonstrated high transient transfection efficiencies and were able to achieve genome integration and generate stable CHO-K1 cells. 

The transfection efficiency of Vero cells was low compared to the other cell lines (Figure 3e). This was also reflected in the stable transfections with the transfecting agents and resulted in the transduction experiments not being feasible. 

## 4. Discussion

Industrial-scale recombinant protein production still relies on the transient plasmid transfection of mammalian cells or on time-consuming and tedious stable cell line generation. Both these processes comprise several bottlenecks such as high costs for plasmid preparation and packaging agents, as well as inefficient and variable transfection processes. Furthermore, the transfection agents have cytotoxic effects on the expressing cells and do not work optimally at high cell densities. We evaluated the use of the BacMam system based on baculoviruses for efficient gene delivery into various mammalian cell lines as it offers cost-effective and robust transduction processes and straightforward scalability. To compare the baculovirus transduction and protein expression in mammalian cells to conventional transfection agents, we constructed an expression cassette encoding the human ACE2 receptor, C-terminally fused to eGFP, to measure the efficiency and the cell-specific gene expression levels. The protein was chosen due to its large size and the complexity of its various post-translational modifications. As displayed in Figure 3a,c,e, we were able to induce protein expression in the three cell lines HEK293-6E, CHO-K1, and Vero. It can clearly be seen that transduction with baculovirus was superior to the transfection agents and achieved higher percentages of GFP-positive cells. In particular, the Vero cell line, which is generally more difficult to transfect with chemical methods [3], showed transduction efficiencies of up to 80% with baculoviral gene transfer. This phenomenon can be explained by the mechanisms behind transfection and transduction. While transfection relies, for example, on positive charges or particle uptake to transfer the genetic material into the host cell, viral particles engage with the cell surface, most likely via gp64, and enter the cell via endocytosis (reviewed in [32]). Additionally, it is important to mention that the transduction with baculovirus has a higher degree of reproducibility and is less prone to variations as can be noticed by the smaller standard deviation (Figure 3a,c,e). This is because the virus preparations do not need to undergo inefficient packaging processes in silico compared to transfection agents, which are prone to variations, even in standardized procedures.

Apart from the efficiency, we evaluated the gene expression level per cell (Figure 3b,d,f). The expression levels of baculovirus transduced cells were generally lower compared to the transfection methods. This might be explained by the fact that the gene dosage delivered by baculoviruses is lower compared to plasmid transfection. While a huge amount of DNA, representing around 500.000 copies per cell, is applied in chemical transfections, the MOIs, meaning single virus particles per cell, are between 10 and 500. Additionally, after the entry of the virus into the cells, the capsid is transferred into the nucleus where the transcription process is started. As the viral DNA is foreign to the host cell, self-defense mechanisms can be activated, and the host enzymes start modifying and thereby silence it [23,24,25]. However, we also addressed this issue and successfully showed that the addition of transcription enhancers compensates for this problem by inhibiting the deacetylation of histones, leading to an elevated gene expression level reaching or even surpassing the levels achieved by chemical transfection procedures (Figure 5).

In bench scale, baculovirus production with Sf9 cells is performed by repeatedly infecting insect cells with the obtained supernatants from infected insect cells. The final working stock is in an insect cell medium and contains a variety of contaminants such as metabolites and chromatin. Depending on the concentration of the viral stock, relatively high volumes need to be added to the mammalian cells to achieve high MOIs. The possible toxicity of the viral stock is one of the suspected reasons why an increase in MOI did not necessarily result in increased protein expression (Figure 2d and Figure 3b,d,f). To test the feasibility of the BacMam technology, a large-scale baculovirus production was performed in a 3.5 L bioreactor. Compared to the bench-scale virus production, a medium lacking FBS, higher cell densities, and a longer production period was chosen for fermentation. A reduced cell viability was observed in the bioreactor. Therefore, the baculovirus was purified using ultracentrifugation, which resulted in the removal of toxic compounds such as chromatin and empty viral particles. The superiority of the purified baculovirus was demonstrated in experiments shown in Figure 6. An increase in MOI was toxic when unpurified baculovirus was used, while purified virus even at MOI of 100 still resulted in higher transduction efficiency as well as expression per cell.

Transient protein expressions usually result in less robust and profitable processes and require efficient plasmid purification and transfection steps. Therefore, the use of stable cell lines is often desirable for large-scale recombinant protein production. In our study, stable transfection was more successful when baculovirus transduction was used instead of the chemical transfection agents. Although the gene dosage delivered is lower in the BacMam system, more cells are being reached. Still, gene expression in transduced cells can be detected, indicating that the transport of the target genes to the nucleus is much more efficient; in contrast, most of the plasmids do not reach the nucleus. By addressing a higher number of cells and bringing more DNA to the nucleus, the chances for a recombination event increase markedly.

The expression of multi-subunit products like AAVs or VLPs still relies on conventional transfection procedures. For example, the production of AAVs used for gene therapy applications requires a triple transfection process. The preparation of three separate plasmids poses an economic burden and increases process variability as the transfection process can be very inefficient. The stable expression of AAV proteins is not applicable due to their inherent toxicity. An alternative could be the baculovirus, as its genome is more than 100 kb in size and has the capacity to carry large cassettes of recombinant DNA [11]. On the other hand, the transfection efficiency of plasmids decreases with increasing size; this effect is not known for transduction. Furthermore, for the production of recombinant virus particles, an array of proteins with different functions is needed to be produced in the cell. The expression level of the various proteins needs to be harmonized and is of importance. Furthermore, maximum protein production is not always desirable as protein quality attributes such as folding or glycosylation might be of higher priority for suitable bioactivity of the therapeutic product. Since the transduction efficiency is higher than most transfecting agents, but the expression level per individual cell is lower, the protein quality might be increased. Moreover, the higher number of viable and transduced cells might account for similar protein yields, but this still remains to be investigated with soluble products. Another advantage of baculoviral transduction is its robustness in processes. The expression data shown in Figure 3 exhibit higher reproducibility compared to transfection, as the standard deviation is much lower for transduction procedures. Overall, our study presents a comprehensive comparison of plasmid transfection to baculovirus transduction for recombinant protein production in three different mammalian cell lines, and the summary is depicted in Table 3.

## Figures and Tables

**Table 1:** Primer sequences used for cloning of pACEBac-1-ACE2-GFP.

Name	Sequence (5′-3′)
23.29 ACE2_GFP golden gate FWD	ggctaccacctgctgacattgattattgactagttattaatagtaatc
23.30 ACE2_GFP golden gate REV	ggctaccacctgcgggctggataagatacattgatgagtttg
23.31 pACEBac1 golden gate FWD	ggctaccacctgcgggctccactgcttgagcctagaag
23.32 pACEBac1 golden gate REV	ggctaccacctgctgaccaatgtcatactagtgtttaaactcgctac

**Table 2:** Number of successful stable transfections in relation to the number of transfections for six different transfecting agents and baculovirus transduction.

Cell Line	PEI	Lipofectamine	Fugene	Calcium Phosphate	Attractene	Polyfect	Baculovirus
HEK293-6E	0/3	0/3	0/3	0/3	1/3	0/3	3/3
CHO-K1	2/5	2/5	1/5	0/5	2/5	0/5	3/3
Vero	0/4	0/4	0/4	0/4	0/4	0/4	n.d. ^1^

^1^ n.d. = not defined.

**Table 3:** Comparison of baculovirus transduction and transfecting agents.

	Baculovirus Transduction	Conventional Transfection
Time to obtain recombinant constructs	1–2 weeks	1–2 weeks
Time for gene transfer agent preparation	propagation of virus 3 weeks	big amount of plasmid up to 1 week
Storage conditions	4 °C	−20 °C
Cost of cultivation of bacteria or insect cells	low to medium	low
BSL1 cell culture facility	yes	yes
Delivery efficiency (various cell lines)	high	low to high
Large cargo capacity	yes	no
Protein yield	comparable	
Stable cell line development	yes (higher probability)	yes

## Data Availability

The data will be available upon request.
